# Association of Bariatric Surgery With Risk of Fracture in Patients With Severe Obesity

**DOI:** 10.1001/jamanetworkopen.2020.7419

**Published:** 2020-06-10

**Authors:** Syed I. Khalid, Philip A. Omotosho, Anna Spagnoli, Alfonso Torquati

**Affiliations:** 1 Department of Surgery, Rush University Medical Center, Chicago, Illinois

## Abstract

**Importance:**

Given the complex relationship between body mass index, body composition, and bone density and the correlative nature of the studies that have established the prevailing notion that higher body mass indices may be protective against osteopenia and osteoporosis and, therefore, fracture, the absolute risk of fracture in patients with severe obesity who undergo either Roux-en-Y gastric bypass (RYGB) or sleeve gastrectomy (SG) compared with those who do not undergo bariatric surgery is unknown.

**Objective:**

To assess the rates of fractures associated with obesity and compare rates between those who do not undergo bariatric surgery, those who undergo RYGB, and those who undergo SG.

**Design, Setting, and Participants:**

In this retrospective multicenter cohort study of Medicare Standard Analytic Files derived from Medicare parts A and B records from January 2004 to December 2014, patients classified as eligible for bariatric surgery using the US Centers of Medicare & Medicaid criteria who either did not undergo bariatric surgery or underwent RYGB or SG were exactly matched in a 1:1 fashion based on their age, sex, Elixhauser Comorbidity Index, hypertension, smoking status, nonalcoholic fatty liver disease, hyperlipidemia, type 2 diabetes, osteoporosis, osteoarthritis, and obstructive sleep apnea status. Data were analyzed from November to December 2019.

**Exposures:**

RYGB or SG.

**Main Outcomes and Measures:**

The primary outcome measured in this study was the odds of fracture overall based on exposure to bariatric surgery. Secondary outcomes included the odds of type of fracture (humerus, radius or ulna, pelvis, hip, vertebrae, and total fractures) based on exposure to bariatric surgery.

**Results:**

A total of 49 113 patients were included and were equally made up of 16 371 bariatric surgery–eligible patients who did not undergo weight loss surgery, 16 371 patients who had undergone RYGB, and 16 371 patients who had undergone SG. Each group consisted of an equal number of 4109 men (25.1%) and 12 262 women (74.9%) and had an equal distribution of ages, with 11 780 patients (72.0%) 64 years or younger, 4230 (25.8%) aged 65 to 69 years, 346 (2.1%) aged 70 to 74 years, and 15 (0.1%) aged 75 to 79 years. Patients undergoing RYGB were found to have no significant difference in odds of fractures compared with bariatric surgery–eligible patients who did not undergo surgery. Patients undergoing undergone SG were found to have decreased odds of fractures of the humerus (odds ratio [OR], 0.57; 95% CI, 0.45-0.73), radius or ulna (OR, 0.38; 95% CI, 0.25-0.58), hip (OR, 0.49; 95% CI, 0.33-0.74), pelvis (OR, 0.34; 95% CI, 0.18-0.64), vertebrae (OR, 0.60; 95% CI, 0.48-0.74), or fractures in general (OR, 0.53; 95% CI, 0.46-0.62). Compared with patients undergoing SG, patients undergoing RYGB had a significantly greater risk of total fractures (OR, 1.79; 95% CI, 1.55-2.06) and humeral fractures (OR, 1.60; 95% CI, 1.24-2.07).

**Conclusions and Relevance:**

In this cohort study, bariatric surgery was associated with a reduced risk of fracture in bariatric surgery–eligible patients. Sleeve gastrectomy might be the best option for weight loss in patients in which fractures could be a concern, as RYGB may be associated with an increased fracture risk compared with SG.

## Key Points

**Question:**

Does severe obesity confer a risk of fracture that could be modified with bariatric surgery?

**Findings:**

In this cohort study of 49 113 bariatric surgery–eligible patients covered by Medicare, patients who were eligible for bariatric surgery but did not undergo surgery had an equal risk of fracture compared with patients undergoing Roux-en-Y gastric bypass but had a greater risk of all site-specific fractures and fractures overall compared with those who underwent sleeve gastrectomy. Among 32 742 patients undergoing bariatric surgery, patients who underwent sleeve gastrectomy were found to be at a significantly reduced risk of developing a humeral fracture or fracture in general compared with those undergoing Roux-en-Y gastric bypass.

**Meaning:**

Bariatric surgery may be associated with lower odds of fracture in patients with severe obesity, and sleeve gastrectomy might be the best option for bariatric surgery in patients in which fractures could be a concern.

## Teaser

This cohort study compares the rates of fractures associated with obesity among bariatric surgery–eligible patients who did not undergo surgery, patients who underwent Roux-en-Y gastric bypass, and patients who underwent sleeve gastrectomy.

## Introduction

Bariatric surgery has increasingly become common as obesity has become a widespread concern in much of the high-income world.^1,2,3,4,5^ These interventions have been shown to be associated with lasting and substantial weight loss, correction and protection from obesity-related conditions, and substantial benefits in quality of life and longevity.^2,3,6,7,8,9,10,11^ Among obesity-related conditions, bariatric surgery has been demonstrated to reduce the burden of metabolic and cardiovascular diseases, migraines, and obesity-related risk of some cancers.^8,12,13,14,15,16,17,18^ There is a large body of literature reporting an observational association between higher body mass index (BMI, calculated as weight in kilograms divided by height in meters squared) and higher bone mineral density (BMD) that has implied that high BMI has protective effects on the skeleton and has led to the inference that loss of excessive body weight may result in decreases in BMD.^11,19,20,21,22^ To this end, bariatric surgery might therefore result in a decreased BMD and serve as a contributor to potentially higher rates and risks of fracture. Furthermore, surgical weight loss approaches that alter the fundamental patterns of alimentary absorption, like Roux-en-Y gastric bypass (RYGB), may serve to hasten this risk and have been associated with the development of metabolic bone disease, resulting in higher bone turnover and long-term declines, disruptions, and deterioration in bone density and bone microarchitecture.^22,23,24,25,26,27,28,29,30^

However, the notion that obesity protects from fracture has been challenged.^31^ Recent studies have reported heightened rates of fracture among those with greater levels of central obesity and in postmenopausal women with obesity.^32,33^ Those studies challenge the value of BMD as a surrogate measure to assess bone fragility in individuals with obesity and furthermore raise questions on the general notion that bariatric surgery is detrimental to the skeleton because it lowers BMD while pointing out the need of long-term studies to assess actual bone fragility after metabolic and weight management surgery. Fracture risk after bariatric surgery has been scarcely studied, and data are contradictory, reporting either no increased risk or limited increased risk. Those inconsistencies are likely due to the wide difference in matching parameters for controls, limited follow-up after surgery, a limited number of participants, and differences in surgical procedures.^23,24,25,26,27,34^ Given the complex relationship between body composition, bone density, and bone fragility as well as the correlative nature of the studies that have established the prevailing notion that higher BMIs may be protective against osteopenia, osteoporosis, and, therefore, fracture, here we explored the absolute risk of fracture in patients with severe obesity who did not undergo bariatric surgery, those who underwent surgical interventions with both restrictive and malabsorptive features (RYGB), and those who underwent surgical interventions with less malabsorptive features (sleeve gastrectomy [SG]).

## Methods

### Data Source

This study followed the Strengthening the Reporting of Observational Studies in Epidemiology (STROBE) reporting guideline. Longitudinal Medicare Standard Analytic Files containing 100% of inpatient and outpatient facility records billed to Medicare derived from Medicare parts A and B from January 2004 to December 2014 were retrospectively analyzed. Patients were defined as eligible for bariatric surgery based on the US Centers of Medicare & Medicaid Services (CMS) criteria for the use of bariatric surgery in adults: BMI of 40 or greater, more than 100 pounds overweight, or BMI of 35 or greater and at least 1 or more obesity-related comorbidities, including type 2 diabetes (T2D), hypertension, obstructive sleep apnea (OSA) and other respiratory disorders, nonalcoholic fatty liver disease (NAFLD), osteoarthritis, lipid abnormalities, gastrointestinal disorders, or heart disease using *International Classification of Diseases, Ninth Revision *(*ICD-9*) diagnosis codes. These criteria, preoperative management, surgical management and procedure, and postoperative management recommendations have remained relatively unchanged throughout the study period and to date.^28^ Patients with a billed interaction at any time during their available Medicare insurance history pertaining to cancer, transplant, end-stage kidney disease, previous gastric operations, gastric banding procedures, or fractures prior to undergoing bariatric surgery were excluded. Patients within this population who underwent RYGB or SG were identified based on *ICD*-*9* procedure codes and *Current Procedural Terminology* codes (eTables 1 and 2 in the Supplement). Patients within this population who did not undergo bariatric surgery were defined as eligible for bariatric surgery. The study was approved by the Rush University Medical Center Institutional Review Board with a waiver of patient informed consent, as the nature of this analysis posed minimal risk to participating individuals and the data were presented in aggregate to minimize any risks of loss of confidentiality of medical data.

### Comorbidities

Demographic data for aggregate records included sex and age. *ICD-9-CM* diagnosis codes were used to identify comorbidities (eTable 1 in the Supplement). Comorbidities were noted as follows: hypertension, smoking status, NAFLD, hyperlipidemia, T2D, osteoporosis, osteoarthritis, postmenopausal status, and OSA. Comorbidities were accessed as a given patient having a billable health care interaction during the CMS bariatric surgery assessment period of 1 year prior to surgery. Age was reported as the age of the patient at the time of surgery. The strategy to use claims-based algorithms to identify patient BMIs has been explored previously and has been demonstrated to be most efficacious in patients who are obese; furthermore, since the institution of Meaningful Use requirements instituted by CMS, BMI reporting has since further improved.^29,30^

### Outcome Definition

The primary outcome measured in this study was the odds of fracture overall based on exposure to bariatric surgery over a 3-year period. Secondary outcomes included the odds of site-specific fractures (humerus, radius or ulna, pelvis, hip, vertebrae, and total fractures) based on exposure to bariatric surgery. Fractures were defined using *ICD*-*9* codes to identify humeral, radial and ulnar, pelvic, hip, and vertebral fractures (eTable 2 in the Supplement). This strategy to use claims-based algorithms to identify fractures has been demonstrated to have a high positive predictive value for these kinds of fractures and have been previously used in this manner to study fracture risk.^32,33,35^

### Statistical Analysis

Descriptive statistics were calculated for age, sex, comorbidities, and postoperative complications to compare the characteristics of bariatric surgery–eligible patients not undergoing bariatric surgery, those who underwent RYGB, and those who underwent SG. Patient populations were exactly matched in a 1:1 fashion based on age, sex, Elixhauser Comorbidity Index, hypertension, smoking status, NAFLD, hyperlipidemia, T2D, osteoporosis, osteoarthritis, and OSA. χ^2^ Tests were then calculated to compare categorical variables, including age ranges, sex, and comorbidity status (hypertension, smoking status, NAFLD, hyperlipidemia, T2D, osteoporosis, osteoarthritis, and OSA). Analysis of variance was used for quantitative variables (Elixhauser Comorbidity Index). Odds ratios (ORs) were calculated to compare fracture events based on bariatric surgery eligibility and whether the patient underwent RYGB or SG. Kaplan-Meier survival analysis of fractures following either RYGB or SG were plotted within 3 years following surgery. Log-rank testing was performed to compare fracture risk between patients undergoing RYGB and SG. Significance levels were adjusted using Bonferroni corrections, and significance was set at a 2-tailed *P* value less than .008. Data were analyzed using R statistical software version 3.42 (The R Foundation).

## Results

### Descriptive Characteristics

**Figure 1.**
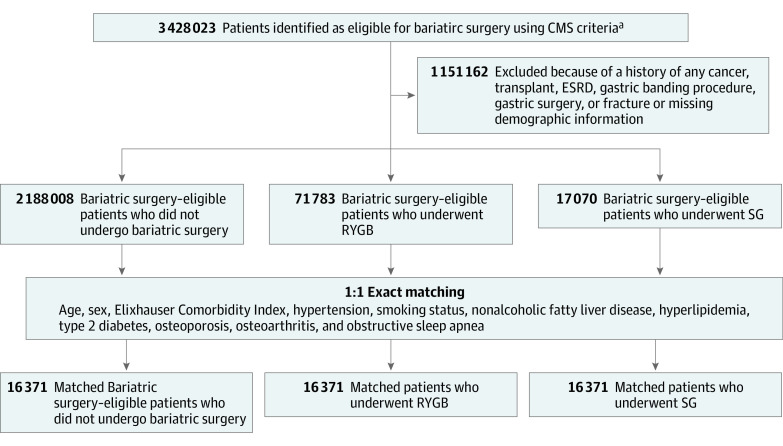
Patient Selection Procedure CMS indicates US Centers of Medicare & Medicaid Services; ESRD, end-stage renal disease; RYGB, Roux-en-Y gastric bypass; SG, sleeve gastrectomy. ^a^Eligibility for bariatric surgery was defined as a body mass index (calculated as weight in kilograms divided by height in meters squared) of 40 or greater, more than 100 pounds overweight, or body mass index of 35 or greater and at least 1 or more obesity-related comorbidities, including type 2 diabetes, hypertension, obstructive sleep apnea and other respiratory disorders, nonalcoholic fatty liver disease, osteoarthritis, lipid abnormalities, gastrointestinal disorders, or heart disease.

A total of 3 428 023 patients were identified as eligible for bariatric surgery based on the criteria outlined by CMS between January 2004 and December 2014. Following exclusion of patients with missing demographic information or a history of cancer, transplant, end-stage kidney disease, gastric operations, or gastric banding procedures, 2 188 008 patients were identified as eligible for bariatric surgery but did not undergo either RYGB or SG, while 71 783 bariatric surgery–eligible patients were found to have undergone RYGB and 17 070 were found to have undergone SG as a weight loss intervention (Figure 1). The descriptive characteristics of the total population are summarized in eTable 3 in the Supplement.

The matched population analyzed in this study contained 49 113 patients, which were equally represented by 16 371 bariatric surgery–eligible patients who did not undergo weight loss surgery, 16 371 patients who underwent RYGB, and 16 371 patients who underwent SG. The demographic distribution and comorbidity status of these patients are summarized in Table 1.

**Table 1.** Descriptive Characteristics of Bariatric Surgery–Eligible Patients and Patients Who Underwent RYGB or SG

Parameters	No. (%)				*P* value
	Total (N = 49 113)	Bariatric surgery–eligible patients (N = 16 371)	RYGB (n = 16 371)	SG (n = 16 371)	
Age, y					
≤64	35 340 (72.0)	11 780 (72.0)	11 780 (72.0)	11 780 (72.0)	>.99
65-69	12 690 (25.8)	4230 (25.8)	4230 (25.8)	4230 (25.8)	
70-74	1038 (2.1)	346 (2.1)	346 (2.1)	346 (2.1)	
75-79	45 (0.1)	15 (0.1)	15 (0.1)	15 (0.1)	
Sex					
Male	12 327 (25.1)	4109 (25.1)	4109 (25.1)	4109 (25.1)	>.99
Female	36 786 (74.9)	12 262 (74.9)	12 262 (74.9)	12 262 (74.9)	
Elixhauser Comorbidity Index, mean (SD)	6.2 (2.9)	6.2 (2.9)	6.2 (2.9)	6.2 (2.9)	>.99
Comorbidity					
Hypertension	35 241 (71.8)	11 747 (71.8)	11 747 (71.8)	11 747 (71.8)	>.99
Smoking	15 123 (30.8)	5041 (30.8)	5041 (30.8)	5041 (30.8)	>.99
NAFLD	336 (0.7)	112 (0.7)	112 (0.7)	112 (0.7)	>.99
Hyperlipidemia	27 918 (56.8)	9306 (56.8)	9306 (56.8)	9306 (56.8)	>.99
Osteoporosis	1869 (3.8)	623 (3.8)	623 (3.8)	623 (3.8)	>.99
Osteoarthritis	12 870 (26.2)	4290 (26.2)	4290 (26.2)	4290 (26.2)	>.99
OSA	22 200 (45.2)	7400 (45.2)	7400 (45.2)	7400 (45.2)	>.99
T2D	23 361 (47.6)	7787 (47.6)	7787 (47.6)	7787 (47.6)	>.99

Abbreviations: NAFLD, nonalcoholic fatty liver disease; OSA, obstructive sleep apnea; RYGB, Roux-en-Y gastric bypass; SG, sleeve gastrectomy; T2D, type 2 diabetes.

Each group consisted of 4109 men (25.1%) and 12 262 women (74.9%) and had an equal distribution of ages, with 11 780 patients (72.0%) aged 64 years or younger, 4230 (25.8%) aged 65 to 69 years, 346 (2.1%) aged 70 to 74 years, and 15 (0.1%) aged 75 to 79 years. Bariatric surgery–eligible patients, patients who underwent RYGB, and patients who underwent SG had mean (SD) Elixhauser Comorbidity Indices of 6.2 (2.9; *P* > .99). Rates of hypertension (71.8%), smoking status (30.8%), hyperlipidemia (56.8%), OSA (45.2%), T2D (47.6%), NAFLD (0.7%), osteoporosis (3.8%), and osteoarthritis (26.2%) were exactly matched and so were the same among bariatric surgery–eligible patients and patients undergoing either RYGB or SG (Table 1).

### Rates and Risk of Fracture

#### Bariatric Surgery–Eligible Patients vs Patients Undergoing RYGB or SG

A total of 1382 patients (2.8%) in the matched population were found to experience a fracture. Of the fractures experienced overall, the greatest number were vertebral fractures (522 [1.1%]). Patients with obesity eligible for bariatric surgery (562 [3.4%]) and patients undergoing RYGB (523 [3.2%]) were found to have similar rates of fractures, whereas patients undergoing SG (297 [1.8%]) experienced significantly lower rates of fracture overall at 3 years following surgery (*P* < .001) (Table 2).

**Table 2.** Rates of Fracture in Patients Who Underwent Bariatric Surgery vs Bariatric Surgery–Eligible Patients Who Did Not at 3 Years Following Surgery

Fracture	No. (%)				*P* value
	Total (N = 49 113)	Bariatric surgery–eligible patients (n = 16 371)	RYGB (n = 16 371)	SG (n = 16 371)	
Humerus	422 (0.9)	170 (1.0)	155 (0.9)	97 (0.6)	<.001^a^
Radius or ulna	176 (0.4)	77 (0.5)	70 (0.4)	29 (0.2)	<.001^a^
Pelvic	86 (0.2)	38 (0.2)	35 (0.2)	13 (0.1)	<.001^a^
Hip	176 (0.4)	69 (0.4)	73 (0.4)	34 (0.2)	<.001^a^
Vertebral	522 (1.1)	208 (1.3)	190 (1.2)	124 (0.8)	<.001^a^
Total	1382 (2.8)	562 (3.4)	523 (3.2)	297 (1.8)	<.001^a^

Abbreviations: RYGB, Roux-en-Y gastric bypass; SG, sleeve gastrectomy.

^a^ Significance was set at a Bonferroni-corrected *P* value less than .008.

There were no significant differences between bariatric surgery–eligible patients and those who underwent RYGB in the odds of experiencing a fracture overall (OR, 0.95; 95% CI, 0.84-1.07), humeral fracture (OR, 0.91; 95% CI, 0.73-1.13), radial or ulnar fracture (OR, 0.90; 95% CI, 0.66-1.26), pelvic fracture (OR, 0.92; 95% CI, 0.58-1.46), hip fracture (OR, 1.06; 95% CI, 0.76-1.47), or vertebral fracture (OR, 0.91; 95% CI, 0.75-1.11) (Table 3). Compared with bariatric surgery–eligible patients who did not undergo surgery, those who underwent SG had lower odds of fractures of the humerus (OR, 0.57; 95% CI, 0.45-0.73), radius or ulna (OR, 0.38; 95% CI, 0.25-0.58), hip (OR, 0.49; 95% CI, 0.33-0.74), pelvis (OR, 0.34; 95% CI, 0.18-0.64),vertebrae (OR, 0.60; 95% CI, 0.48-0.74), or in general (OR, 0.53; 95% CI, 0.46-0.62) (Table 3).

**Table 3.** Odds of Fracture in Patients Who Underwent RYGB or SG vs Bariatric Surgery–Eligible Patients Who Did Not at 3 Years Following Surgery

Fracture	Odds Ratio (95% CI)	
	RYGB vs bariatric surgery–eligible patients	SG vs bariatric surgery–eligible patients
Humerus	0.91 (0.73-1.13)	0.57 (0.45-0.73)^a^
Radius or ulna	0.90 (0.66-1.26)	0.38 (0.25-0.58)^a^
Pelvic	0.92 (0.58-1.46)	0.34 (0.18-0.64)^a^
Hip	1.06 (0.76-1.47)	0.49 (0.33-0.74)^a^
Vertebral	0.91 (0.75-1.11)	0.60 (0.48-0.74)^a^
Total	0.95 (0.84-1.07)	0.53 (0.46-0.62)^a^

Abbreviations: RYGB, Roux-en-Y gastric bypass; SG, sleeve gastrectomy.

^a^ *P* < .008.

#### RYGB vs SG

**Figure 2.**
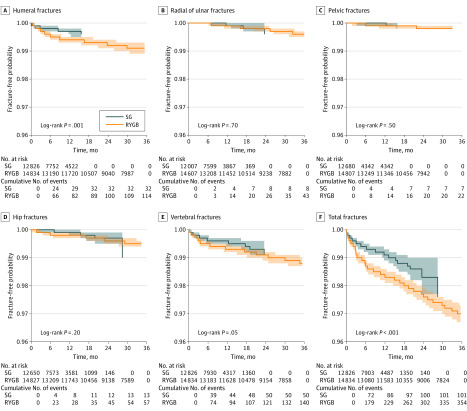
Site-Specific and Total Fracture Risk at 3 Years Following Bariatric Surgery in Patients Undergoing Roux-en-Y Gastric Bypass (RYGB) vs Sleeve Gastrectomy (SG)

When comparing the rates of developing fractures within 3 years following surgery, patients undergoing RYGB had a significantly greater risk of total fractures and fractures of the humerus compared with those undergoing SG (total: OR, 1.79; 95% CI, 1.55-2.06; humerus: OR, 1.60; 95% CI, 1.24-2.07). There were no significant differences in the risk associated with developing fractures of the radius or ulna, pelvis, hip, or vertebrae between patients who underwent RYGB and those who underwent SG (Figure 2).

## Discussion

In this matched cohort analysis of 49 113 bariatric surgery–eligible patients, including 16 371 who did not undergo bariatric surgery, 16 371 who underwent RYGB, and 16 371 who underwent SG, it was found that patients who were eligible for bariatric surgery but did not undergo surgery had a similar odds of fracture as patients undergoing RYGB at 3 years following surgery. Furthermore, patients who underwent SG had significantly lower odds of all site-specific fractures and fractures overall compared with bariatric surgery–eligible patients. Among patients undergoing bariatric surgery, patients who underwent SG were found to be at a significantly reduced risk of developing a humeral fracture or fracture in general.

The present study provides valuable clinical information to the field of bariatrics by providing, for the first time to our knowledge, specific analysis of the risk of fracture among patients who undergo bariatric surgery compared with patients who are eligible for bariatric surgery but do not. Furthermore, this study establishes a timeline for this deferential risk in patients who undergo RYGB compared with patients who undergo SG. In addition, this study demonstrates challenging information to the long-supported idea in medicine that patients with higher BMI (and so patients who are obese) experience a protective benefit against osteoporosis and fractures by illustrating a potential protection against fracture in patients who undergo bariatric surgery. Furthermore, SG was found to be more protective against fracture compared with RYGB.

### Fracture Risk in Bariatric Surgery and Obesity

Increasing BMI and obesity have been long associated with higher BMD and lower incidences of fracture.^7,22,28,36,37,38,39,40,41,42,43^ Very recently, the Look AHEAD trial,^37,38,39^ a multicenter randomized clinical trial that was designed to determine whether intentional weight loss reduces cardiovascular morbidity and mortality in overweight individuals with T2D, suggested that even relatively small percentages of weight loss (6% to 9%) are associated with significant reductions in BMD and with increased risk of hip, pelvis, and upper arm fracture. Given that most studies that report total body weight loss following RYGB and SG have reported a mean percentage of total weight loss of more than 25% and 18%, respectively, at 5 years, the notion that this kind of major weight loss itself results in an increase in the fracture risk profile of patients would reasonably follow.^7,17,44,45,46,47,48^ Emerging data have started to challenge the notion that obesity is protective against all fractures and has supported that patterns of fat deposition (body fat sites and ratios of visceral fat to subcutaneous fat) and body compositions (muscle mass) may have a greater influence on BMD and fracture risk profiles than BMI alone.^49,50,51,52,53,54,55,56,57^

The present study assessed the risk of fractures of the humerus, radius or ulna, pelvis, hip, and vertebrae in patients undergoing bariatric surgery and those who were eligible to undergo bariatric surgery but did not and found that obesity conferred a significantly greater risk of all fracture types and fractures overall compared with patients undergoing SG and similar risks for all fracture types and fractures overall compared with patients undergoing RYGB, providing evidence for a potential protective effect of weight loss against the risk of fractures. In the case of RYGB, a malabsorptive mechanism and complex bone metabolism changes may serve to further complicate this relationship. This does not mean that BMD does not change in these patients; in fact, several studies have described long-term changes in BMD following weight loss surgery as measured by BMD scans and markers of bone turnover, but this further underscores a more multifaceted and multifactorial risk profile for fractures in patients with severe obesity.^58,59,60,61,62,63,64,65,66,67,68,69,70^

### Risk of Fracture in RYGB vs SG

Surgical weight loss approaches that alter the fundamental patterns of alimentary absorption, like RYGB, have been shown to hasten this risk and have even been associated with the development of metabolic bone disease, resulting in higher bone turnover and long-term declines, disruptions, and deterioration in bone density and bone microarchitecture. However, it is important to note that many of the clinical studies that have studied postoperative fracture risk and BMD changes following RYGB have a number of limitations; very few studies provide nonsurgical controls or detail age-related changes or measurement drifts, several studies lack preoperative measurements of BMD or provide baseline measures of bone turnover, and only a limited number of studies quantify rates of BMD loss or risk of fracture between different bariatric procedures.^59,60,71,72,73^ Despite these shortcomings in clinical studies and challenges in bone density imaging, most evidence in the literature to date suggests that surgical weight loss procedures may have long-term and persistent negative effects that might differ by surgical approach. Specifically, higher levels and rates of BMD loss and fracture risk in patients undergoing RYGB have been postulated to be caused in part by the malabsorptive implications and associated changes in alimentary-associated hormones ghrelin, glucagon-like peptide 1, and peptide YY, changes in estradiol, leptin, visfatin, resistin, and adiponectin, and changes in bile acid metabolism.^74,75,76,77^

Unfortunately, there is a paucity of evidence or information regarding both the independent risks of fracture imparted by SG or comparing bone loss with fracture risk in patients undergoing RYGB, with the very few studies available either providing conflicting information with regards to BMD loss or being underpowered.^72,78,79,80^ These limited clinical data, when interpreted in the context of the limited animal studies comparing bone loss seen following RYGB vs SG, seem to show that the rate of bone loss following SG is less than that observed in RYGB.^81^ The present study, to our knowledge, demonstrates for the first time a comparative increase in fracture risk, albeit demonstrated to be lower than their bariatric equivalents, in patients undergoing RYGB vs SG. Specifically, this increased risk was found to be significant in fractures overall and humerus fractures but was not significant when comparing the risk of fractures of the radius or ulna, pelvis, hip, or vertebrae. To this end, it appears that the risk of fracture in patients following SG is less than that observed with RYGB, although additional investigation is required to better elucidate this risk and, further, the expressed relationship between BMD loss and fracture following RYGB and SG.

### Limitations

Administrative data allow access to more medical visits nationwide and longitudinal tracking of these patients through distinct identifiers based on a standardized coding system; however, important limitations in the use of these data must be considered. First, administrative data are intended for financial and administrative use rather than research purposes and therefore may vary in detail and accuracy. Second, administrative data also do not provide qualifiable details on the severity of disease states or patient-reported outcome scores or allow for standardization of treatment protocols or surgeon technique or expertise, which may mask certain confounding factors. Third, specifically for the purposes of this article, administrative data limit the assessment of the specific weight loss a patient may experience as a result of bariatric surgery and so we are unable to directly assess any potential associations between absolute weight loss and fracture risk.

## Conclusions

The generally accepted notion that obesity is protective when considering the risks of fracture may not be as straightforward as previously thought. The relationship between BMI, body composition, and bone density may play an important role when evaluating the risk of fracture in patients with obesity. Severe obesity status alone might be associated with an increased risk of fracture, and there is a role for weight loss surgery in augmenting this risk. Specifically, SG might be the best option for weight loss in patients in whom fractures could be a concern, as RYGB may be associated with an increased fracture risk compared with SG. Additional studies are needed to not only further characterize the risk profile of obesity on rates of fracture but also to access fracture risk and benefits of different surgical weight loss options.

## Notes

Supplement. **eTable 1.** Coding Definitions **eTable 2.** Coding Definitions For Fracture Categories **eTable 3.** Descriptive Characteristics of Bariatric Surgery–Eligible Patients and Patients Who Underwent Roux-en-Y Gastric Bypass or Sleeve Gastrectomy
