# Supplementary material for: Association of Bariatric Surgery With Risk of Fracture in Patients With Severe Obesity
Source: JAMA Netw Open. 2020 Jun 10;3(6):e207419. doi: 10.1001/jamanetworkopen.2020.7419 (PMC7287567; doi:10.1001/jamanetworkopen.2020.7419)
Supplement: Supplement. — eTable 1. Coding Definitions eTable 2. Coding Definitions For Fracture Categories eTable 3. Descriptive Characteristics of Bariatric Surgery–Eligible Patients and Patients Who Underwent Roux-en-Y Gastric Bypass or Sleeve Gastrectomy [file jamanetwopen-3-e207419-s001.pdf]

Supplementary Online Content

Khalid SI, Omotosho PA, Spagnoli A, Torquati A. Association of bariatric surgery with risk of fracture in patients with severe obesity. *JAMA Netw Open*. 2020;3(6):e207419. doi:10.1001/jamanetworkopen.2020.7419

eTable 1. Coding Definitions
eTable 2. Coding Definitions For Fracture Categories
eTable 3. Descriptive Characteristics of Bariatric Surgery–Eligible Patients and Patients Who Underwent Roux-en-Y Gastric Bypass or Sleeve Gastrectomy

This supplementary material has been provided by the authors to give readers additional information about their work.

© 2020 Khalid SI et al. *JAMA Network Open.*

eTable 1. Coding Definitions

**Category**	**ICD-9 Codes, (CPT Codes)**
**Osteoporosis**	*73300, 73301, 73302, 73303, 73309*
**Hypertension**	*4010, 4011, 4019*
**Smoking Status**	*3051, V1582*
**Obstructive Sleep Apnea**	*32723*
**Hyperlipidemia**	*272.0, 272.1, 272.2, 272.3*
**Osteoarthritis**	*71500, 71510, 71509, 71516, 71530, 71531, 71590, 71504*
**Type 2 Diabetes**	*25000, 25002, 25010, 25012, 25020, 25022, 25030, 25032, 25040, 25050, 25042, 25052, 25060, 25062, 25070, 25072, 25080, 25082, 25090, 25092*
**NAFLD**	*5715*
**BMI >35**	*V8535, V8536, V8537, V8538, V8539, V8541, V8542, V8543, V8544, V8545, 27801*
**Postmenopausal Status**	*6279, 6278, 25631, 6272, 6273, V074, V4981*
**Sleeve Gastrectomy**	4382, (43775)
**Roux-en-Y Gastric Bypass**	*4438, 4439, (43644, 43645, 43846, 43847)*

© 2020 Khalid SI et al. *JAMA Network Open.*

eTable 2. Coding Definitions For Fracture Categories

Fracture	ICD-9 Code
Humerus	*812.00, ICD-9-D-81201, ICD-9-D-81202, ICD-9-D-81203, ICD-9-D-81209, ICD-9-D-8121, ICD-9-D-81210, ICD-9-D-81211, ICD-9-D-81212, ICD-9-D-81213, ICD-9-D-81219, ICD-9-D-8122, ICD-9-D-81220, ICD-9-D-81221, ICD-9-D-81230, ICD-9-D-81231, ICD-9-D-8124, ICD-9-D-81240, ICD-9-D-81241, ICD-9-D-81242, ICD-9-D-81243, ICD-9-D-81244, ICD-9-D-81249, ICD-9-D-81250, ICD-9-D-81251, ICD-9-D-81252, ICD-9-D-81253, ICD-9-D-81254, ICD-9-D-81259, ICD-9-D-73311*
Radius or Ulnar	*ICD-9-D-73312, ICD-9-D-81300, ICD-9-D-81301, ICD-9-D-81302, ICD-9-D-81303, ICD-9-D-81304, ICD-9-D-81305, ICD-9-D-81306, ICD-9-D-81307, ICD-9-D-81308*
Pelvic	*ICD-9-D-73398, ICD-9-D-8080, ICD-9-D-8081, ICD-9-D-8082, ICD-9-D-8083, ICD-9-D-80841, ICD-9-D-80842, ICD-9-D-80843, ICD-9-D-80844, ICD-9-D-80849, ICD-9-D-80851, ICD-9-D-80852, ICD-9-D-80853, ICD-9-D-80854, ICD-9-D-80859, ICD-9-D-8088, ICD-9-D-8089*
Hip	*ICD-9-D-73314, ICD-9-D-73396, ICD-9-D-82000, ICD-9-D-82001, ICD-9-D-82002, ICD-9-D-82003, ICD-9-D-82009, ICD-9-D-82010, ICD-9-D-82011, ICD-9-D-82012, ICD-9-D-82013, ICD-9-D-82019, ICD-9-D-82020, ICD-9-D-82021, ICD-9-D-82022, ICD-9-D-82030, ICD-9-D-82031, ICD-9-D-82032, ICD-9-D-8208, ICD-9-D-8209*
Vertebral	*ICD-9-D-8052, ICD-9-D-8054, ICD-9-D-8058, ICD-9-D-73313*

© 2020 Khalid SI et al. *JAMA Network Open.*

eTable 3. Descriptive Characteristics of Bariatric Surgery–Eligible Patients and Patients Who Underwent Roux-en-Y Gastric Bypass or Sleeve Gastrectomy

**Parameters**	**Total (N=2,276,861)**	**Bariatric Surgery-Eligible Patients (N=2,188,008)**	**Roux-en-Y Gastric Bypass (N=71,783)**	**Sleeve Gastrectomy (N=17,070)**	**p-Value**
**Age**					*<.001*[a]
64 and under, n (%)	*965,845 (42.4)*	*899,878 (41.1)*	*54,308 (75.7)*	*11,659 (68.3)*	
65-69, n (%)	*613,060 (26.9)*	*594,866 (27.2)*	*13,812 (19.2)*	*4,382 (25.7)*	
70-74, n (%)	*337,852 (14.8)*	*333,916 (15.3)*	*3,027 (4.2)*	*909 (5.3)*	
75-79, n (%)	*199,847 (8.8)*	*199,190 (9.1)*	*542 (0.8)*	*115 (0.7)*	
80-84, n (%)	*104,213 (4.6)*	*104,136 (4.8)*	*77 (0.1)*	-	
85 and over, n (%)	*56,039 (2.5)*	*56,022 (2.6)*	*17 (0.02)*	-	
**Sex**					*<.001*[a]
Male, n (%)	*799,056 (35.1)*	*777,521 (35.5)*	*17,161 (23.9)*	*4,374 (25.6)*	
Female, n (%)	*1,477,805 (64.9)*	*1,410,487 (64.5)*	*54,622 (76.1)*	*12,696 (74.4)*	
**Comorbidities**					
DM (II), n (%)	*982,336 (43.1)*	*938,136 (42.9)*	*36,107 (50.3)*	*8,093 (47.4)*	*<.001*[a]
NAFLD, n (%)	*27,731 (1.2)*	*26,539 (1.2)*	*957 (1.3)*	*235 (1.4)*	*.003*[a]
Osteoarthritis, n (%)	*472,497 (20.8)*	*448,567 (20.5)*	*19,370 (27.0)*	*4,560 (26.7)*	*<.001*[a]
Hypertension, n (%)	*1,482,607 (65.1)*	*1,421,105 (64.9)*	*49,293 (68.7)*	*12,209 (71.5)*	*<.001*[a]
Smoking Status, n (%)	*552,334 (24.3)*	*528,219 (24.1)*	*18,702 (26.1)*	*5,413 (31.7)*	*<.001*[a]
Hyperlipidemia, n (%)	*1,133,070 (49.8)*	*1,084,805 (49.6)*	*38,549 (53.7)*	*9,716 (56.9)*	*<.001*[a]
Osteoporosis, n (%)	*138,157 (6.1)*	*133,623 (6.1)*	*3,739 (5.2)*	*795 (4.7)*	*<.001*[a]
OSA, n (%)	*440,826 (19.4)*	*406,588 (18.6)*	*26,429 (36.8)*	*7,809 (45.7)*	*<.001*[a]
[a]*Significant Values (p < 0.008)*					

© 2020 Khalid SI et al. *JAMA Network Open.*
